# Case Report: Early experience with hypoxia-targeted SBRT-based partial tumor irradiation in large and bulky tumors

**DOI:** 10.3389/fonc.2025.1697888

**Published:** 2025-12-02

**Authors:** Jose Penagaricano, Justyn Y. Nakashima

**Affiliations:** Department of Radiation Oncology, Moffitt Cancer Center, Tampa, FL, United States

**Keywords:** SBRT-PATHY, immunomodulation, abscopal effect, by-stander effect, spatially fractionated radiation therapy

## Abstract

Large and bulky tumors possess poor prognostic characteristics that make tumor control difficult. Stereotactic body radiotherapy (SBRT)-based partial tumor irradiation targeting the hypoxic segment (SBRT-PATHY) has been described as a spatially fractionated radiotherapy technique that induces both bystander and abscopal effects by sparing the peritumoral immune microenvironment, thus minimizing the negative effects of radiation-induced lymphopenia. These case reports aim to present our early experience with four cases utilizing SBRT-PATHY at our institution.

## Introduction

Management of advanced bulky tumors in the metastatic setting is complex. These are tumors that are unresectable and present with aggressive features, making them difficult to control with standard radiotherapy (RT) techniques. Stereotactic body radiotherapy (SBRT) is commonly employed in definitive and oligometastatic settings due to its ability to deliver ablative doses with high conformality (1). However, the anatomical and spatial proximity between bulky tumors and organs at risk (OARs) presents a planning challenge, as it is often not possible to meet surrounding tissue tolerance, potentially resulting in patient toxicity.

Immunomodulatory effects of RT, although not clearly defined yet, have shown promise by not only inducing primary target responses but also by inducing bystander and abscopal effects. The abscopal effect refers to a systemic antitumor response where localized irradiation of the primary target not only impacts the targeted tumor but also leads to regression of distant, nonirradiated lesions (2). This immunologic phenomenon is observed or amplified with the use of hypofractionated regimens or SBRT in combination with immunotherapy. Similarly, the bystander effect involves radiation-induced changes in nearby, nonirradiated cells due to intercellular signaling, potentially contributing to both therapeutic benefit and toxicity (3, 4).

Spatially fractionated radiotherapy (SFRT) techniques, such as GRID or Lattice, have shown promising results in large, bulky tumors secondary to immunomodulation and direct tumor effects while minimizing toxicity (5). Thus, combining SFRT and immunotherapy could improve the outcome of large and bulky tumors (5). Another form of SFRT, SBRT-based partial tumor irradiation targeting hypoxic segments (SBRT-PATHY), exploits immune-related bystander and abscopal effects (6, 7). Available literature shows that selectively targeting the hypoxic segment of a large tumor with ablative dosing induces nontargeted effects, resulting in increased tumor control and minimized side effects. A key aspect of SBRT-PATHY is the intentional sparing of the peritumoral immune microenvironment (PIM), which contains critical locoregional immune components such as antigen-presenting cells (APCs), lymphocytes, and proinflammatory cytokines. By preserving this region, the treatment helps maintain an immunostimulatory environment, thereby enhancing the immunomodulatory impact of therapy and promoting both bystander and abscopal effects (3, 7). This contrasts with traditional conventional or palliative RT efforts that target the immune microenvironment, thus causing immunosuppression (3). This can result in poor tumor control due to RT-induced lymphopenia secondary to immune cell depletion in the tumor microenvironment.

This work aims to present our early experience with four cases of SBRT-PATHY for large metastatic bulky tumors.

## Methods

The clinical information from our first four cases of SBRT-PATHY was extracted from the patients’ medical records with prior IRB approval [MCC23903; Pro00089137].

Patient selection included metastatic or oligometastatic patients with a gross tumor volume (GTV) of at least 5 cm in any one dimension. Histologies considered to be nonradiosensitive were allowed (i.e., sarcoma, squamous cell carcinoma, adenocarcinoma, melanoma). The intent could be either palliative or curative. For this work, prior immunotherapy was allowed (i.e., failure of immunotherapy).

Planning (6) of SBRT-PATHY is as shown below.

### SBRT-PATHY planning regions of interest

**Figure 1:**
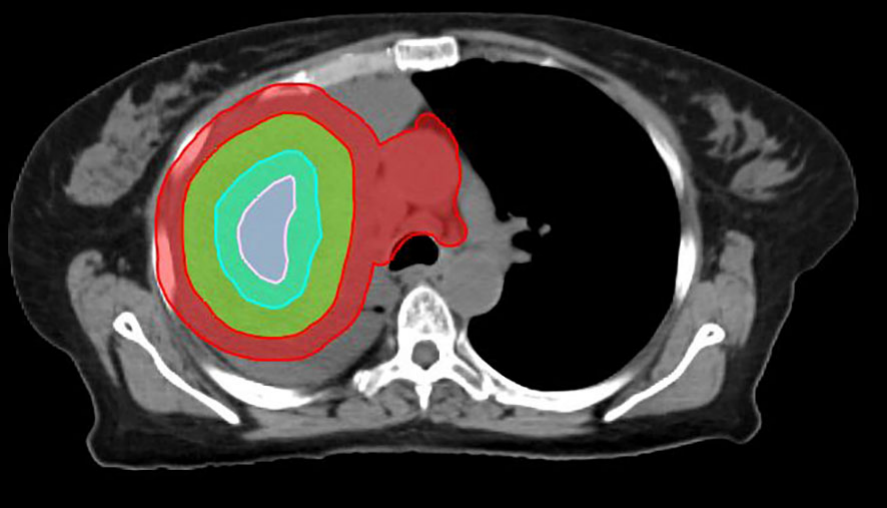
Planning regions of interest defined in SBRT-PATHY for a lung cancer case. Due to the size of the mass, two BTVs were created (BTV36Gy and BTV30Gy in three fractions). The contours in green, pink, sky blue, and red represent the GTV, BTV36, BTV30, and PIM, respectively.

SBRT-PATHY planning requires identifying specific regions within and around the tumor to deliver targeted radiation effectively while preserving surrounding immune structures (**Figure 1**). The main planning regions are outlined below:

1. GTV: the GTV is the tumor as seen on diagnostic imaging (e.g., MRI, Computed Tomography (CT), Positron Emission Tomography – Computed Tomography (PET-CT)).
2. Bystander treatment volume (BTV): the BTV is defined based on contrasted CT or FDG-PET-CT. It corresponds to the central hypoxic region of the GTV and typically represents approximately one-third of the GTV. This can be achieved by using Boolean operators until approximately 30% of the GTV is obtained. Alternatively, if hypoxic imaging is available, the BTV is defined as the hypoxic region plus a 5-mm margin; if PET-CT is used, it is defined as the volume with a standardized uptake value (SUV) ≤ 3 or 30% of the maximum standardized uptake value (SUV_max_). If contrasted CT is used for contouring the GTV, the average GTV-to-BTV ratio is approximately 3:1. The prescribed radiation dose is delivered to the BTV, which represents approximately 30% of the GTV (bulky tumor) (6). Theoretically, any hypoxia-specific imaging agent can be used to outline the BTV, including Cu-64 ATSM and 18F-MISO. On average, the BTV represents approximately 30% of the GTV, regardless of the imaging modality used for contouring.
3. PIM: tumor-associated immune cells are located at the surface of the GTV. The PIM is defined as a 1-cm expansion of the GTV, with the GTV subtracted (PIM = [1 cm + GTV] − GTV). The PIM also includes any nearby lymph nodes or lymphatic organs, which are added manually.

### Prescription and constraints

Doses and constraints were set for each planning region to ensure effective tumor coverage while minimizing exposure to surrounding normal tissues. Details are provided below:

1. BTV: prescription to the BTV is one to three fractions of 10–12 Gy each, with a maximum dose limited to 14.5 Gy per fraction.
2. PIM: *D*_max_ (maximum dose) < 5Gy per fraction, *D*_min_ (minimum dose) < 1 Gy per fraction, and *D*_average_ (average dose) < 3Gy per fraction.
3. OAR: follow published guidelines, such as TG-101 (16).

**Figure 2:**
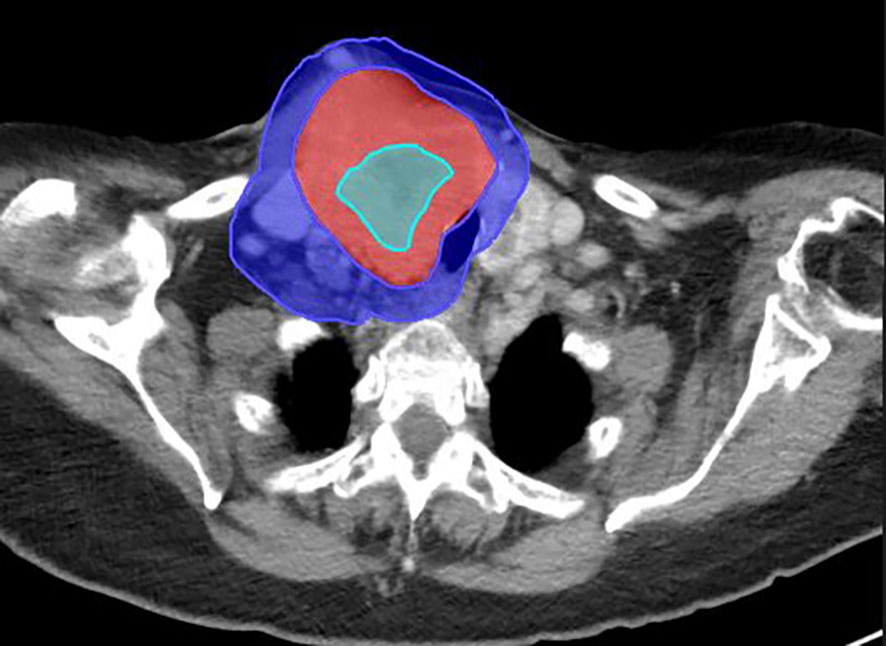
Large right neck mass from metastatic follicular thyroid cancer on the diagnostic CT scan. Mass measures 6.9 cm × 8.5 cm × 10.5 cm. The BTV (sky blue), GTV (red), and PIM (blue) are shown on this diagnostic scan.

**Figure 3:**
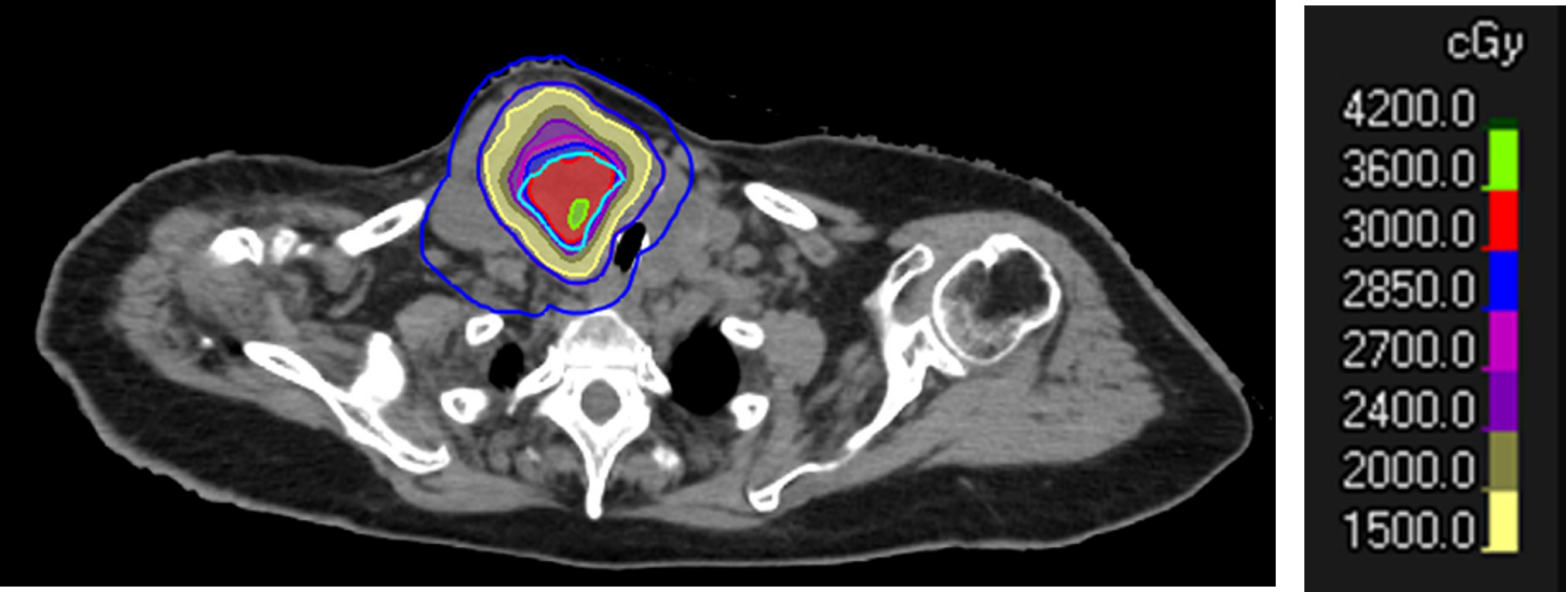
Dose distribution of SBRT-PATHY to a large right neck mass from follicular thyroid cancer. Note the sparing of the peritumoral immune microenvironment (PIM) in blue. The prescription was set to 30 Gy in three fractions to the BTV30Gy. The BTV contour is shown in sky blue, and the GTV in red (outer red line).

**Figure 4:**
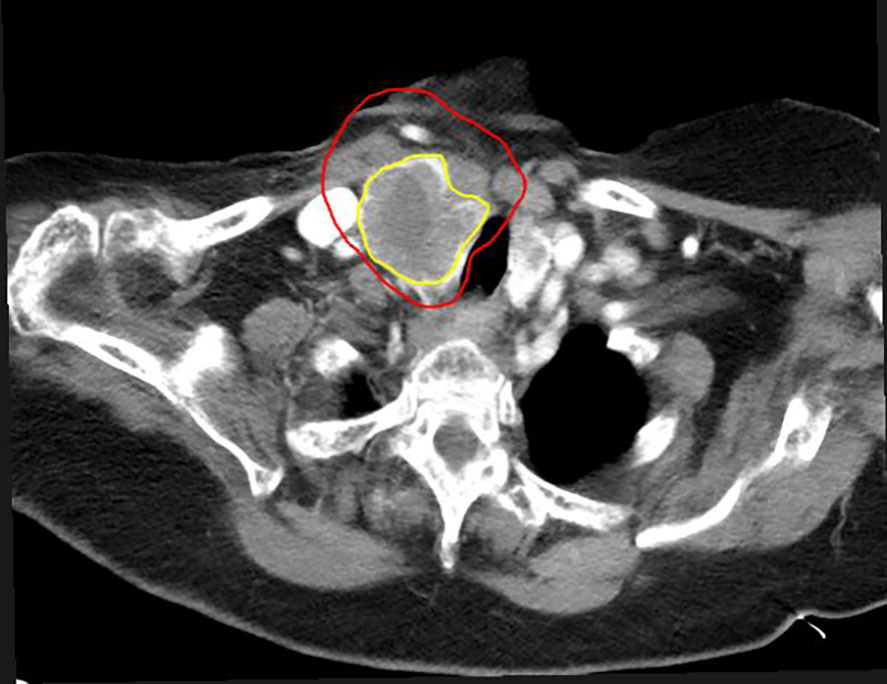
Six months after SBRT-PATHY. Note the reduction in tumor volume in a case of metastatic thyroid cancer. Tumor measures 3.9 cm × 7.0 cm × 4 cm (82% reduction in volume compared to pre-SBRT-PATHY imaging). The initial GTV is shown in red, and the residual GTV in yellow.

A typical tumor treated with SBRT-PATHY, along with its dose distribution and 6-month follow-up imaging, is shown in **Figures 2**–**4**, respectively (case 3).

## Results

### Case 1 (E.S.)

A 55-year-old woman with metastatic large-cell neuroendocrine carcinoma of the right lung presented with a 7-cm right lung mass and prominent mediastinal lymphadenopathy.

She was started on carboplatin, etoposide, and pembrolizumab; however, pembrolizumab was discontinued due to immune-mediated colitis. The patient was managed with infliximab and vedolizumab while continuing carboplatin and etoposide.

She subsequently enrolled in a clinical trial for DLL3-directed Chimeric Antigen Receptor T-cell (CAR-T) therapy, receiving fludarabine and cyclophosphamide followed by CAR-T infusion.

Two months postinfusion, the mass increased to 10.9 cm. Due to disease progression, she was removed from the CAR-T trial. Lurbinectedin was then initiated but discontinued because of poor tolerance.

The patient received SBRT-PATHY, with 30 Gy delivered to the BTV30Gy and a simultaneous integrated boost to BTV36Gy of 36 Gy in three fractions. She reported no acute toxicity during treatment or during the short follow-up period (6 weeks).

Two weeks posttreatment, imaging showed a reduction of the right lung tumor to 7.8 cm, although there was a mild increase in mediastinal lymphadenopathy. One month post-SBRT-PATHY, the patient was started on tarlatamab.

Approximately 1.5 months after SBRT-PATHY, she developed right-sided facial droop and slurred speech. Imaging confirmed an embolic stroke and pulmonary embolism, along with progression of the right lung mass to 13.1 cm, mediastinal lymphadenopathy, and new bilateral pulmonary nodules.

The patient subsequently chose to enroll in hospice care.

### Case 2 (J.H.)

A 64-year-old woman presented with cutaneous metastatic squamous cell carcinoma involving the left inguinal lymph nodes, measuring 10.8 cm × 8.7 cm × 9.9 cm.

She was started on cemiplimab. Follow-up imaging revealed progression of the left inguinal mass, with compression of the superficial and deep femoral neurovascular bundles.

She received SBRT-PATHY to the left inguinal mass to BTV30Gy in three fractions, with no toxicity reported during treatment or the short follow-up period (4 months). At her 1-month follow-up, her pain had completely resolved. Restaging CT demonstrated a significant reduction in the dominant left inguinal nodal mass, now measuring 5.8 cm × 3.4 cm × 7.9 cm. Additional sites of lymphadenopathy in the mediastinum, retroperitoneum, pelvis, and iliac chain had also decreased in size.

Two months after completing radiation therapy, the patient was readmitted with an occlusive deep vein thrombosis in the left lower extremity. She underwent thrombectomy, angioplasty, and stenting. Subsequent follow-up visits demonstrated continued positive clinical and radiographic response to treatment, with no evidence of disease recurrence or progression.

Four months after completing radiation therapy, imaging showed a continued reduction of the treated left inguinal mass. However, a new disease appeared and was progressing at other sites.

### Case 3 (A.G.)

An 89-year-old woman with metastatic follicular thyroid cancer was initially diagnosed after noticing right-sided neck enlargement. CT of the neck showed a large, complex mass in the right lobe of the thyroid gland, measuring 10.5 cm and causing tracheal compression.

The patient underwent an endocrinology evaluation prior to starting lenvatinib.

She received SBRT-PATHY to the right thyroid mass to BTV30Gy in three fractions, with no significant toxicity other than grade 2 radiation dermatitis. No additional toxicities were reported during treatment or the short follow-up period (2 months). Lenvatinib was started soon after.

Two-and-a-half-month follow-up CT of the neck showed continued reduction in the size of the mass. However, new lymphadenopathy was observed in the right neck, supraclavicular fossa, and axilla.

Although there was a favorable tumor response in the neck, the presence of new adenopathy with an atypical pattern of spread raised concern for a more aggressive tumor. The patient is therefore currently being evaluated for treatment with lenvatinib plus pembrolizumab.

### Case 4 (T.C.)

A 62-year-old woman with metastatic squamous cell carcinoma of the lung presented with a 3.8 cm × 3.8 cm × 2.9 cm heterogeneously enhancing mass in the right supraclavicular region.

The patient began chemoimmunotherapy with carboplatin, paclitaxel, ipilimumab, and nivolumab. One-month follow-up CT showed disease progression, with enlargement of the right supraclavicular mass.

She received SBRT-PATHY to the right supraclavicular mass to BTV30Gy in three fractions, with no significant toxicity during treatment or the short follow-up period (2 weeks). Subsequent imaging revealed significant disease progression, including extensive liver metastases. Given the continued progression and her limited response to treatment, she opted for hospice care. Imaging 6 weeks after SBRT-PATHY showed further progression of the right supraclavicular mass.

## Discussion

The proposed mechanism underlying the therapeutic efficacy of SBRT-PATHY involves both direct cytotoxic effects and systemic immune modulation. Partial tumor irradiation has been associated with upregulation of apoptosis-inducing factor (AIF) not only within the irradiated portion of the tumor but also at distant, untreated tumor sites. Furthermore, increased infiltration of CD3+/CD8+ cytotoxic T lymphocytes has been observed, particularly within the PIM, suggesting localized activation of antitumor immunity (6). Interestingly, although this lymphocyte proliferation was localized to the PIM, abscopal sites showed upregulation of cell death-inducing cytokines, such as interferon-gamma (IFN-γ), interleukin-6 (IL-6), tumor necrosis factor-alpha (TNF-α), and TNF-related apoptosis-inducing ligand (TRAIL), indicating systemic immune activation (6).

Although abscopal responses following RT are rare, preclinical studies suggest that partial tumor irradiation—especially approaches that intentionally spare the immune-rich peritumoral regions—can shift the immune balance from a suppressive to a stimulatory state, thereby enhancing systemic antitumor responses. In addition, irradiating the hypoxic tumor segment has been shown to augment bystander effects through increased release of damage-associated molecular patterns (DAMPs) and hypoxia-induced immunogenic modulation (8).

Clinical data reinforce these observations. In a phase II trial involving 60 patients with unresectable, bulky non-small cell lung cancer (NSCLC), SBRT-PATHY demonstrated superior outcomes compared with standard-of-care chemotherapy and conventional palliative radiotherapy. Specifically, the 1-year overall survival and cancer-specific survival rates were 75% and 90%, respectively, in the SBRT-PATHY arm—compared with 60% for both endpoints with standard-of-care chemotherapy and 20% with institutional conventional palliative radiotherapy. Tumor control rates for bulky lesions reached 95% with SBRT-PATHY, versus only 20% in both comparator arms. Notably, bystander and abscopal effects were observed in 95% and 45% of SBRT-PATHY-treated patients, respectively, supporting the hypothesis of an immunomodulatory mechanism (7).

Additional studies have reported favorable outcomes with partial tumor irradiation targeting hypoxic regions. Massaccesi et al. (9) administered single-fraction 10 Gy SBRT to hypoxic tumor segments identified via FDG-PET in patients with recurrent bulky tumors. Among the nine lesions treated, responses included one complete response, two partial responses, and two cases of stable disease, with no acute toxicities recorded. One disease progression was observed 10 weeks after treatment. Similarly, Yan et al. reported a phase I dose-escalation trial in patients with bulky, unresectable solid tumors. The maximum tolerated dose was 18 Gy × 3 fractions, with no grade ≥ 3 toxicities observed among 30 patients. Although details on target definition were limited, high rates of disease control were reported.

Korpics et al. (10) evaluated the combination of SBRT and pembrolizumab in patients with advanced solid tumors. A subset of patients received partial tumor irradiation for tumor volumes > 65 cm^3^, with dose regimens ranging from 10–15 Gy × 3 fractions to 10 Gy × 5 fractions. One-year local control rates were high, at 92.4% overall, including 86.7% for partial and 94.6% for complete tumor irradiation.

The feasibility of SBRT-PATHY using particle therapy (protons or carbon ions) has also been explored. Tubin et al. (11) reported on 11 patients treated with particle-based partial irradiation. At a median follow-up of 6.3 months, overall survival was 64%, with a median survival of 6.7 months. Progression-free survival reached 46%, with an average tumor volume regression of 61%. The abscopal effect was observed in 60% of patients, and the symptom control rate reached 91%.

In our own institutional experience, among four patients treated with SBRT-PATHY, two demonstrated significant treatment responses. Case 2 showed complete resolution of the treated mass, with sustained response at 4 months, while case 3 exhibited an 82% reduction in tumor volume within 2.5 months. However, both cases also showed disease progression at distant, nonirradiated sites. Case 1 initially demonstrated a 50% reduction in tumor volume but subsequently progressed within the treated field. Case 4 did not exhibit any meaningful response to therapy. Notably, no abscopal effects were observed in our small cohort. For cases that received immunotherapy, it is difficult to ascertain whether the tumor response was solely attributable to SBRT-PATHY, as these patients were treated outside of a clinical trial. The observed response may have resulted from immunotherapy, SBRT-PATHY, or a combination of both.

As discussed, the tumor microenvironment is dynamically reshaped after radiotherapy by both immune-stimulatory processes and counter-regulatory pathways. Our cases provide examples of significant tumor volume reduction associated with the bystander effect. One debated outcome of these immunomodulatory interactions is the abscopal effect. Although promising preclinical data and anecdotal clinical observations have highlighted this phenomenon, its reproducibility and true clinical significance remain uncertain, given its rarity and inconsistent association with immunotherapy (12–14). Our institutional experience has similarly reflected this variability.

Unresolved questions remain regarding the optimal radiation dose and fractionation schedule, tumor histology, and potential synergies with immunotherapy that could influence the likelihood of an abscopal response. Additional well-designed mechanistic and prospective clinical studies are needed to better define the circumstances under which abscopal responses occur and to determine how radiotherapy might be optimally integrated with immunomodulatory approaches to consistently leverage this effect for patient benefit. Although our study did not consider the specific timing of immunotherapy, Tubin et al. (15) demonstrated that synchronizing SBRT-PATHY with the oscillating immune response may increase the probability of inducing bystander and abscopal effects.

Other SFRT techniques, such as GRID or Lattice, function similarly to SBRT-PATHY. They exploit the immune environment, inducing bystander and abscopal effects through immunomodulation, in addition to high-dose areas that exert antiangiogenic effects. No current guidelines or standards exist for selecting a specific technique in a given case. Choice of technique currently depends on the physician’s expertise and the patient’s needs, which are discussed during the informed consent process.

Treatment-related toxicity in our series was limited, with only case 3 experiencing grade 2 radiation dermatitis during treatment or the short follow-up period.

## Conclusion

While we did not observe an abscopal effect in our case series, a robust bystander response was evident in our thyroid and squamous cell carcinoma (SQCC) of the skin cases. These findings suggest that SBRT-PATHY, like other SFRT modalities, may help overcome tumor radioresistance by modulating the local immune microenvironment, potentially enhancing both local and distant tumor control. Given this immunomodulatory potential, the combination of SBRT-PATHY with immunotherapy appears to be a rational and promising strategy for the management of large, bulky tumors. Prospective clinical trials are warranted to better define the tumor types and patient populations most likely to benefit from this approach.

## Data Availability

The raw data supporting the conclusions of this article will be made available by the authors, without undue reservation.
